# METTL3 enhances dentinogenesis differentiation of dental pulp stem cells via increasing *GDF6* and *STC1* mRNA stability

**DOI:** 10.1186/s12903-023-02836-z

**Published:** 2023-04-11

**Authors:** Yue Pan, Ying Liu, Dixin Cui, Sihan Yu, Yachuan Zhou, Xin Zhou, Wei Du, Liwei Zheng, Mian Wan

**Affiliations:** 1 State Key Laboratory of Oral Diseases, National Clinical Research Center for Oral Diseases, Department of Pediatric Dentistry, West China Hospital of Stomatology, Sichuan University, Chengdu, Sichuan China; 2 Shenzhen Stomatology Hospital (Pingshan) of Southern Medical University, Shenzhen, Guangdong China; 3 State Key Laboratory of Oral Diseases, National Clinical Research Center for Oral Diseases, Department of Cariology and Endodontics, West China Hospital of Stomatology, Sichuan University, Chengdu, Sichuan China

**Keywords:** Mesenchymal stem cells, Dentinogenesis, Epigenesis, METTL3, RNA stability

## Abstract

**Background:**

The dentinogenesis differentiation of dental pulp stem cells (DPSCs) is controlled by the spatio-temporal expression of differentiation related genes. RNA N6-methyladenosine (m^6^A) methylation, one of the most abundant internal epigenetic modification in mRNA, influences various events in RNA processing, stem cell pluripotency and differentiation. Methyltransferase like 3 (METTL3), one of the essential regulators, involves in the process of dentin formation and root development, while mechanism of METTL3-mediated RNA m^6^A methylation in DPSC dentinogenesis differentiation is still unclear.

**Methods:**

Immunofluorescence staining and MeRIP-seq were performed to establish m^6^A modification profile in dentinogenesis differentiation. Lentivirus were used to knockdown or overexpression of METTL3. The dentinogenesis differentiation was analyzed by alkaline phosphatase, alizarin red staining and real time RT-PCR. RNA stability assay was determined by actinomycin D. A direct pulp capping model was established with rat molars to reveal the role of METTL3 in tertiary dentin formation.

**Results:**

Dynamic characteristics of RNA m^6^A methylation in dentinogenesis differentiation were demonstrated by MeRIP-seq. Methyltransferases (METTL3 and METTL14) and demethylases (FTO and ALKBH5) were gradually up-regulated during dentinogenesis process. Methyltransferase METTL3 was selected for further study. Knockdown of METTL3 impaired the DPSCs dentinogenesis differentiation, and overexpression of METTL3 promoted the differentiation. METTL3-mediated m^6^A regulated the mRNA stabiliy of *GDF6* and *STC1*. Furthermore, overexpression of METTL3 promoted tertiary dentin formation in direct pulp capping model.

**Conclusion:**

The modification of m^6^A showed dynamic characteristics during DPSCs dentinogenesis differentiation. METTL3-mediated m^6^A regulated in dentinogenesis differentiation through affecting the mRNA stability of *GDF6* and *STC1*. METTL3 overexpression promoted tertiary dentin formation in vitro, suggesting its promising application in vital pulp therapy (VPT).

**Supplementary Information:**

The online version contains supplementary material available at 10.1186/s12903-023-02836-z.

## Introduction

The pulp tissue is surrounded by the dentin with the function of sensation, nutrition, defense, restoration and immune regulation [1]. It senses the exogenous stimuli via dentinal tubules. Preserving the pulp vitality contributes maximal benefits for physiological function of teeth. Especially for young permanent teeth, vital pulp is essential for root and apical formation. Responding to external factors, such as microbes, chemicals and trauma, the pulp tissue is capable of secreting dentin matrix and generating tertiary dentin. Vital pulp therapy (VPT), including indirect capping, direct capping and pulpotomy, applies capping agents to isolate external stimulus and induce tertiary dentin formation, which always compromises into unsatisfied long-term prognosis and results in endodontic therapy [2–5]. The dentinogenesis potential of dental pulp stem cells (DPSCs) is the basic biological foundation of VPT, which suggests enhancing DPSCs dentinogenesis differentiation as a strategy to improve clinical prognosis of VPT [6].

The DPSCs dentinogenesis differentiationis is regulated by a range of growth factors and transcriptional regulators, such as BMP, TGF, FGF, Wnt, and the synergistic regulation of signaling pathways [7, 8]. Previous studies reported that BMP2, TGFβ1 and FGF2 enhanced the alkaline phosphatase activity of dental pulp cells, and increased the formation of mineralized nodules [9, 10]. Wnt signal run through the whole process of tooth development and Wnt6 activated JNK signal pathway to promote DPSCs migration and differentiation [11, 12]. The programmed spatiotemporal expression of these key genes depends on the genetics and multi-layered epigenetic mechanisms. [13]

As the most profuse mRNA internal modification in eukaryotic cells, RNA m^6^A modification involves in the post-transcriptional regulation by promoting nuclear processing, maturation, export, activating, translation, and mRNA stability. [13–15] It was reported that reducing the m^6^A modification level inhibit the differentiation of embryonic stem cells, indicating that RNA m^6^A modification took an important place in stem cell proliferation and differentiation [16]. Methyltransferases, including METTL3, METTL14 and WTAP, can form complexes to perform catalytic functions together. METTL3 has been demonstrated to potentiate osteogenic differentiation of bone marrow mesenchymal stem cell (BMSCs) [17]. In DPSCs, METTL3 knockdown interfered cell senescence and apoptosis and impaired root formation [18, 19]. While, the mechanism of METTL3 in regulating the dentinogenesis differentiation in a m^6^A-dependent manner remains unclear.

Therefore, the present study was designed to establish the dynamic profile of RNA m^6^A methylation during DPSC dentinogenesis differentiation, reveal the role and mechanism of METTL3-mediated m^6^A during this process, and explore the potential application of METTL3 in VPT.

## Materials and methods

### Immunofluorescence staining

Eight-week-old adult male C57BL/6 mice were given excessive carbon dioxide for euthanasia, and were sacrificed to collect mandible samples. The whole mandibles were fixed in 4% paraformaldehyde immediately after dissection. The decalcified samples were embedded in paraffin, and were prepared into 4‐μm‐thick sections (model HM 340E; Microm). Antibodies against m^6^A (1:200; Synaptic Systems), goat anti-rabbit AlexaFluor 488 (1:500; Abcam) and DAPI (Solarbio) were used.

### Cell culture, treatment and transfection

DPSCs were isolated from human third molars and cultured based on the previous protocol [6]. The negative control medium (NC) was Dulbecco's modified Eagle's medium (Gibco) with 10% foetal bovine serum (FBS; Gibco) and 100 U/mL penicillin–streptomycin (Hyclone). The dentinogenesis medium (OM) was NC medium supplemented with 10 mmol/L sodium β‐glycerophosphate (Sigma‐Aldrich), 10 nmol/L dexamethasone (Sigma‐Aldrich) and 50 μg/mL l‐ascorbic acid (Sigma‐Aldrich).

As the degree of cell fusion reached 20–30%, the medium was halved and added polybrene with virus. The amount of virus added per hole was calculated according to the formula given by GeneCopoeia and the virus titer [20]. The amount of lentivirus = the number of cells * MOI / lentivirus titer. The MOI used in this experiment was 70.

### Alkaline phosphatase and alizarin red staining

DPSCs were fixed after 7-day cultured, and the ALP staining and quantification was guided by ALP staining kit protocol (Beyotime). The mineralized nodules formation of DPSCs was evaluated by alizarin red S staining (Cyagen) after 14 days cultured.

### Western blotting

DPSCs were lysed by RIPA buffer (50 mmol/L Tris [pH 8], 250 mmol/L NaCl, 0.05% sodium dodecyl sulphate, 0.5% deoxycholate, 1% NP‐40) to collect total proteins. They were isolated on a 10% Bis‐Tris protein gel (1.0 mm) and were transferred to nitrocellulose membrane (Bio‐Rad). Anti-METTL3 (Proteintech), anti-m^6^A (Synaptic Systems) and horseradish peroxidase (HRP)‐conjugated were used. The membranes were detected by a chemiluminescent reagent kit, and were scanned with a GS‐700 imaging densitometer (Bio‐Rad) and then analyzed in ImageJ software (National Institute of Health).

### RNA-Seq and qPCR

DPSCs total RNAs were extracted by TRIzol reagents (Invitrogen) and the RNA-seq experiments (RNA sequencing) were performed by Novogene. Total RNAs were reverse‐transcribed to cDNA with the HiScript III RT SuperMix for qPCR (+ gDNA wiper, Vazyme). Real‐time RT‐PCR was fulfilled with ChamQ Universal SYBR qPCR Master Mix (Vazyme) and an ABI 7900 system (Applied Biosystems). Primers used in the study were listed in the Additional file 1: Table S1. The reactions were normalized to GAPDH and relative gene expression levels were analyzed by ΔΔCt values.

### Methylated RNA immunoprecipitation sequencing (MeRIP-seq)

Total RNAs were also extracted by TRIzol reagents. The mRNAs were enriched with polyA with Oligo-dT magnetic beads and were segmented directly by ultrasound. The segmented mRNA was divided into two parts. One was added with antibodies to capture m^6^A, and was enriched the mRNA fragments with m^6^A methylation. The other was used as input to directly construct a routine transcriptome sequencing library. Antibody of m^6^A was enriched by magnetic beads, and the mRNA fragment with m^6^A was recovered. The routine sequencing libraries were constructed according to the process of transcriptome construction, and were sequenced by Illumina Hiseq X Ten. Peak detection analysis (peak calling) were performed with MeTDiff software.

### GO analysis and KEGG pathway enrichment analysis

The Gene Ontology (GO) project provided a controlled vocabulary for describing gene and gene product attributed in any organism [21–23]. The statistical significance of the differences between the differentially expression list and the GO annotation list was assessed using Fisher's exact test in Bioconductor's top GO. The significance of the GO term enrichment for differentially expressed genes was indicated by the P-value generated by top GO. Genes were mapped to KEGG pathways in pathway analysis [24–26]. The ingenuity pathway analysis was used to perform KEGG enrichment analysis, which linked differentially m^6^A-methylated or expressed mRNAs to biological pathways, and the significance of the pathway correlated to the conditions was represented by the P-value. For the differential m^6^A-methylated sites and differential m^6^A-methylated mRNAs in the pathway, the statistical significance thresholds were established as |FC|≥ 1.5 and P-value ≤ 0.05.

### RNA stability assay

Cells were collected 0 h, 3 h, 6 h, 12 h, and 24 h after cultured with 5 μg/ml actinomycin D (Sigma, USA). Total RNAs were isolated for RT-PCR and all the mRNA levels of target genes were compared with GAPDH levels to normalize.

### Direct pulp capping model

Eight-week-old adult male SD rats were used and anesthetized by intraperitoneally injecting 10% chloral hydrate at a dose of 50 mg/kg. Then, pulpal foramen were produced by a 1 mm-diameter bur on occlusal surfaces of the maxillary first molars, diameter roughly half of the bur. After irrigation and hemostasis, sterile gelatin sponges (GSs) (HU SHI DA) soaked in lentivirus were placed on the exposed pulp. Animals were divided into four groups, treated with GSs without lentiviral (control group), GSs with METTL3-overexpression lentiviral (METTL3 OE group), GSs and iRoot BP plus (Henry Schein)(iRoot group), and GSs with METTL3 overexpression lentiviral and iRoot BP combined capping treatment (METTL3 OE + iRoot group). Then the crown was filled with conventional glass ionomer cement and resins. One molar in each maxillary was left untreated as control. The animals were given excessive carbon dioxide for euthanasia and were sacrificed 30 days after surgery.

### Micro CT

The maxillae were collected in phosphate buffer saline (PBS; Gibco). Micro CT analysis was executed on a VivaCT40 scanner (SCANO) at 75KVp and 6W. Images were captured at the the maxillary first molar and were analyzed with ImageJ software to measure the intensity of tertiary dentin.

### H&E staining

The maxillae were collected and fixed in paraformaldehyde. After embedded in paraffin, the samples were sliced into 4‐μm‐thick sections, and stained with haematoxylin/eosin.

### Statistical analysis

All experiments were repeated at least three times, and the results were presented as means and standard deviation of triplicate measurements. All data were evaluated statistically using Student's t tests or one‐way ANOVA with the Tukey post hoc test.

## Results

### The expression level of RNA m^6^A modification increased during dentinogenesis differentiation

**Fig. 1:**
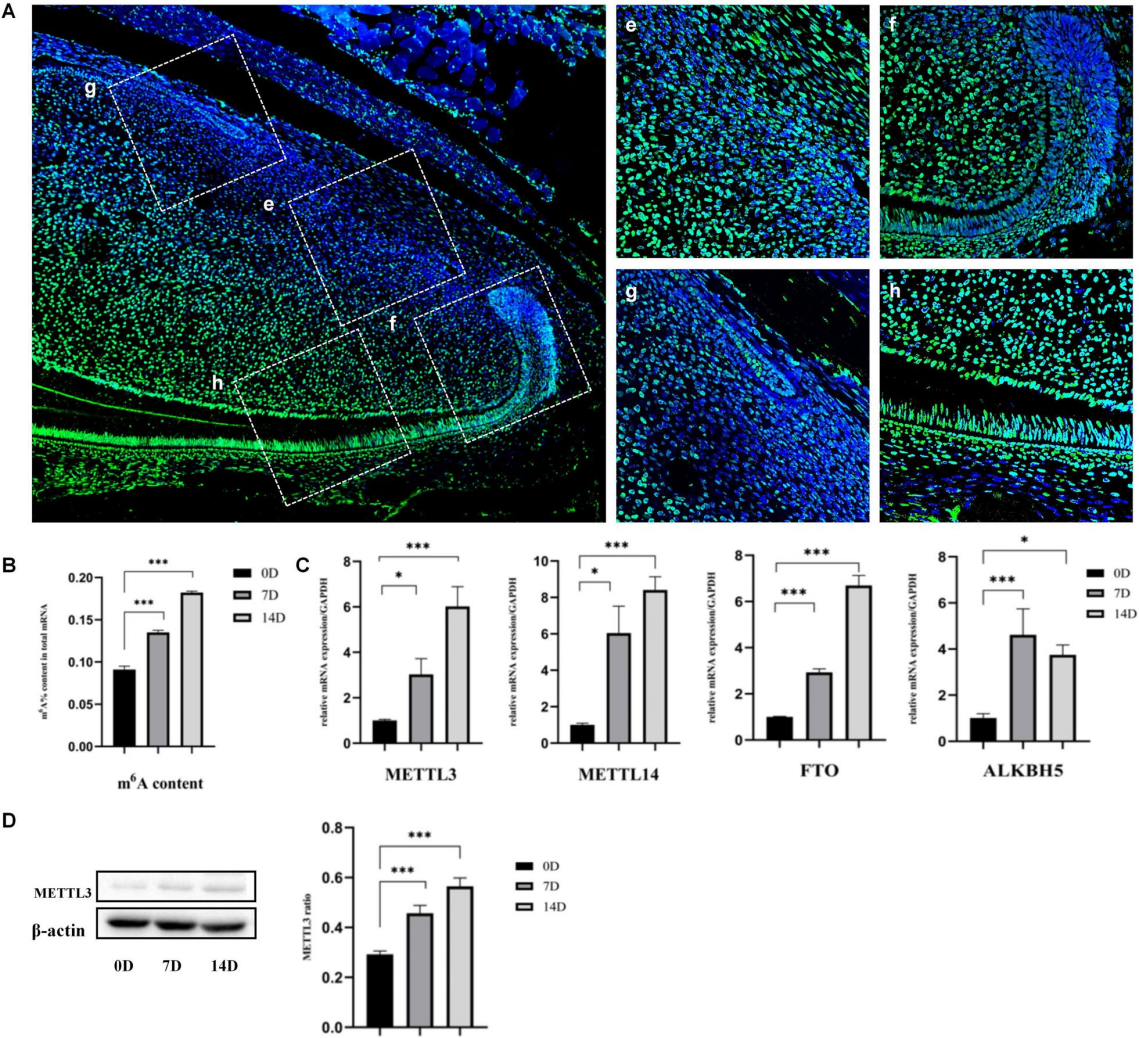
The level of RNA m^6^A modification increased during dentinogenesis differentiation. **A** The expression of m^6^A gradually increased with the dentinogenesis differentiation in mice cervical loop. **e** Stem cells. **f** Transit-amplifying cells in labial cervical loop. **g** Transit-amplifying cells in lingual cervical loop. h)Odontoblasts. **B** The content of RNA m^6^A increased in DPSCs during the dentinogenesis differentiation. **C** Expression levels of RNA m.^6^A methylation-related genes, METTL3, METTL14, FTO, ALKBH5, were measured by real‐time RT‐PCR on 0, 7 and 14 days after mineralization induction (n = 3). **D** Western blotting analysis of METTL3 from DPSCs after mineralization induct for 0, 7 and 14 days. β‐ACTIN served as an internal control. *P < 0.05, ***P < 0.001

Immunofluorescence staining indicated that m^6^A modification was widely present in nucleus of dental mesenchymal cells, including stem cells, transit-amplifying cells (TACs), and odontoblasts. The fluorescence intensity was weakest in the stem cell niche (Fig. 1e), gradually increased in TACs (Fig. 1f, g), and reached the highest in odontoblasts (Fig. 1h), suggesting the level of m^6^A modification gradually increased during dentinogenesis differentiation (Fig. 1A).

To further confirm the change of m^6^A modification, the m^6^A content was measured by colorimetric method. The content of m^6^A was up-regulated during dentinogenesis differentiation in vitro (Fig. 1B). The mRNA levels of methyltransferases (METTL3 and METTL14) and demethylases (FTO and ALKBH5) were detected by RT-PCR. All of them were gradually up-regulated from 0 to 14d mineralization induction (Fig. 1C). The western blotting results also confirm that the expression of METTL3 in DPSCs was gradually up-regulated during the differentiation (Fig. 1D). This dynamic m^6^A modification suggested that m^6^A participated in the regulation of DPSCs dentinogenesis differentiation.

### The RNA m^6^A modification had spatiotemporal specificity in dentinogenesis differentiation

**Fig. 2:**
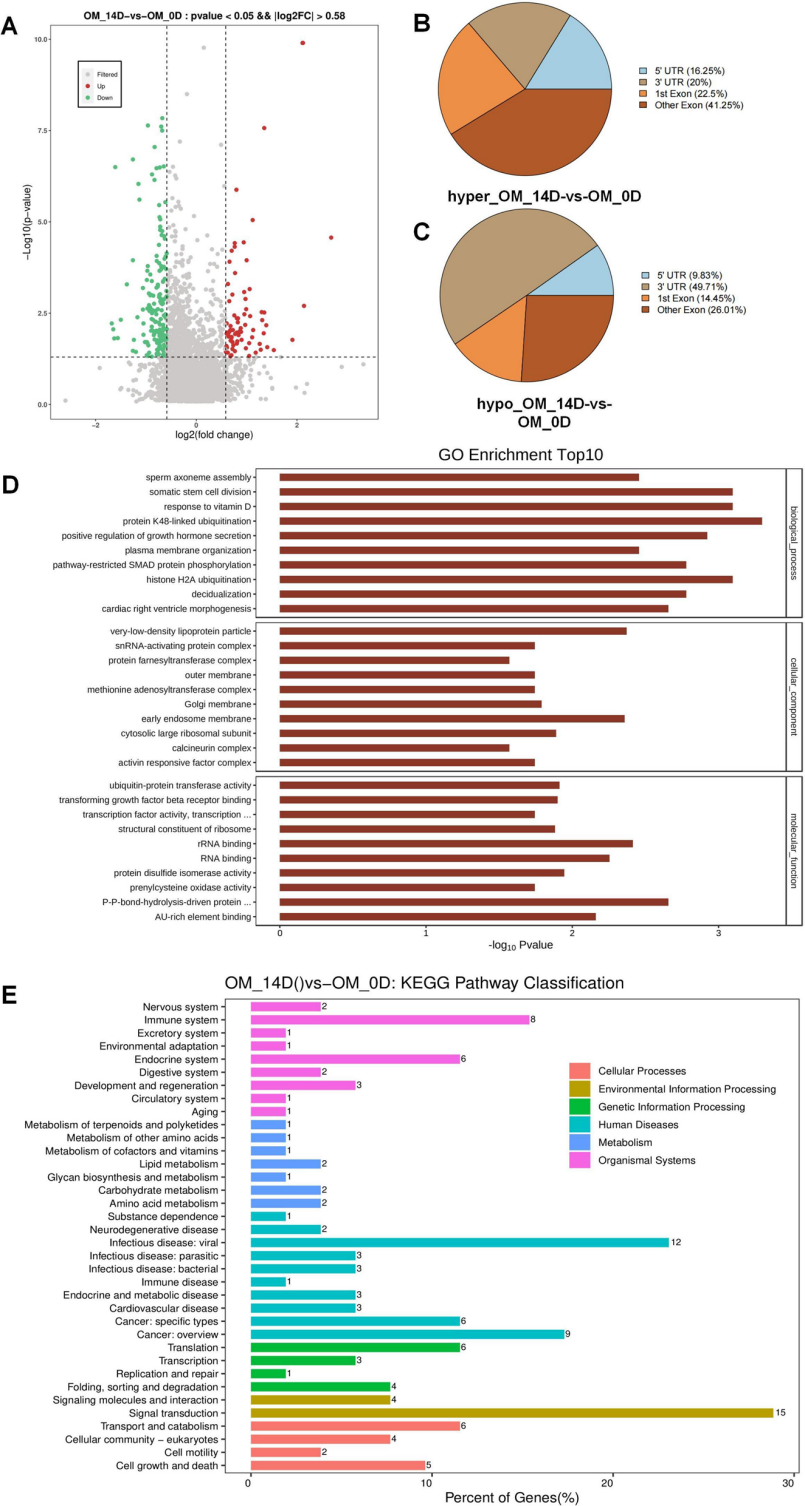
The differential m^6^A-methylated peaks and differential m^6^A methylation distribution in DPSCs (n = 4). **A** The volcano diagram showed the differential m^6^A methylation peaks detected in DPSCs. **B**, **C** Pie diagrams revealed the annotation of hypermethylation and hypomethylation peaks. **D** GO Enrichment Top10. **E** KEGG pathway classification of differential peak related genes

To further explore the dynamic characteristics of RNA m^6^A modification during dentinogenesis differentiation, the RNA m^6^A profiles of DPSCs with both 0d and 14d OM cultured were established by Me-RIP seq. DPSCs cultured for 0d were set as control. Among 253 significantly differential peaks identified, there were 80 hypermethylated peaks with related genes, including *GDF6, MAML3, STC1, UFL1, ZNF14*, and et al. 173 hypomethylated peaks were related to genes, such as *BMP6, FGF10-AS1, MAT2A, OXCT2, and SMAD4* (Fig. 2A). The GO analysis showed that the differential methylated genes were involved in biological process, including protein K48-linked ubiquitination, somatic stem cell division, response to vitamin D, and histone H2A ubiquitination (Fig. 2D). KEGG analysis also demonstrated that differential methylated genes during dentinogenesis differentiation were enriched in pathways of human diseases (45), organismal systems (25), environmental information processing (19), cellular processes (17), genetic information processing (14), and metabolism (10) (Fig. 2E). Peak annotation showed that the hypermethylated peaks were mainly distributed in the other Exon (41.25%), followed by the 1st Exon (22.5%), the 3ʹ-UTR (20%), and the 5ʹ-UTR (16.25%) (Fig. 2B). The hypomethylated peaks were distributed the most in the 3ʹ-UTR (49.71%), followed by the other Exon (26.01%), the 1st Exon (14.45%), and the fewest located in the 5ʹ-UTR (9.83%) (Fig. 2C). These results further confirmed the spatiotemporal pattern of RNA m^6^A modification during DPSC dentinogenesis differentiation, suggesting the potential role of RNA m^6^A modification in the regulation of the differentiation process.

### Joint analysis of MeRIP-seq and RNA-seq indentified hypermethylated and up-transcripted genes

**Fig. 3:**
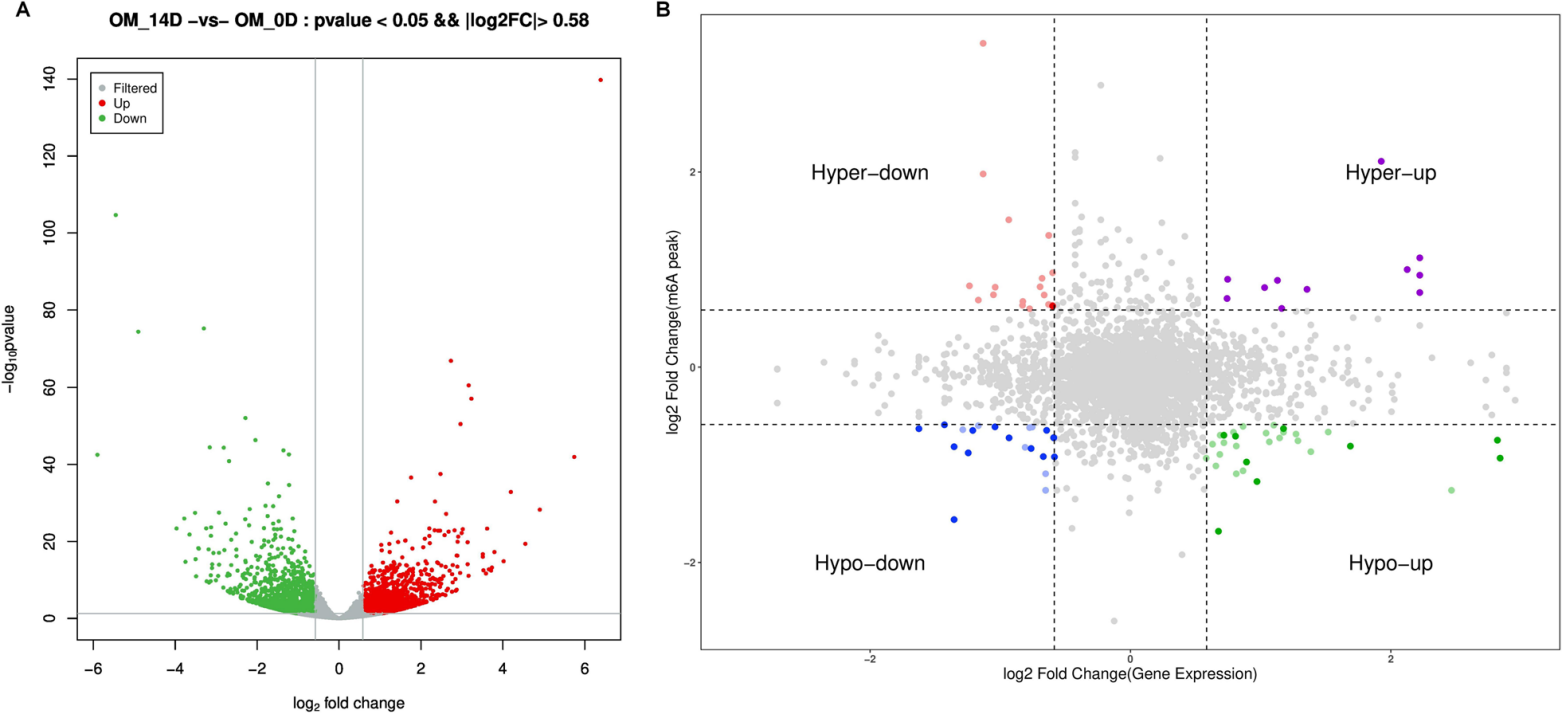
Joint Analysis of MeRIP-seq and RNA-seq (n = 4). **A** The volcano diagram showed the differential expression genes in DPSCs. **B** The combined analysis of differential m^6^A methylation and RNA expression

Differential RNA expression was analyzed with DESeq2 software. Among 1481 up-transcripted genes identified, there were dentinogenesis differentiation-related genes (*BMP2, DSP, EREG, FGF10,* and *SMAD5*) (Fig. 3A), indicating that OM induced DPSCs dentinogenesis differentiation. Besides, METTL3 was up-regulated as well, which was selected for further study in the present study.

Then, joint analysis of Me-RIP seq and RNA-seq were performed to explore the potential mechanism of RNA m^6^A modification in dentinogenesis differentiaiton. The results showed that there were 8 genes with hyper-methylation and up-transcription (*GDF6, MAML3, MICALCL, OXCT2, STC1, UFL1, ZNF441,* and *ZNF804A*), 3 genes with hyper-methylation and down-transcription (*ALB, SERPINA3,* and *ZNF547*), 11 genes with hypo-methylation and up-transcription (*AKAP5, ANKH, CENPP, DGKI, FGF10, HIF1A, NAMPT, NOTCH2NL, SMIM3, TAF9B,* and *ZFC3H1*), and 14 genes with hypo-methylation and down-transcription (*APOL1, C6orf132, CREG1, ENDOD1, FAM46A, LOC102723728, NPIPA1, SOWAHD, SVEP1, TGFB2, TLR3, TMEM47, UHRF1BP1,* and *ZFP36L2*) (Fig. 3B). Considering RNA m^6^A modification increased during DPSCs differentiation, genes with hyper-methylation and up-transcription might function as key components of RNA m^6^A methylation during this process.

### METTL3 involved in the dentinogenesis differentiation in vitro

**Fig. 4:**
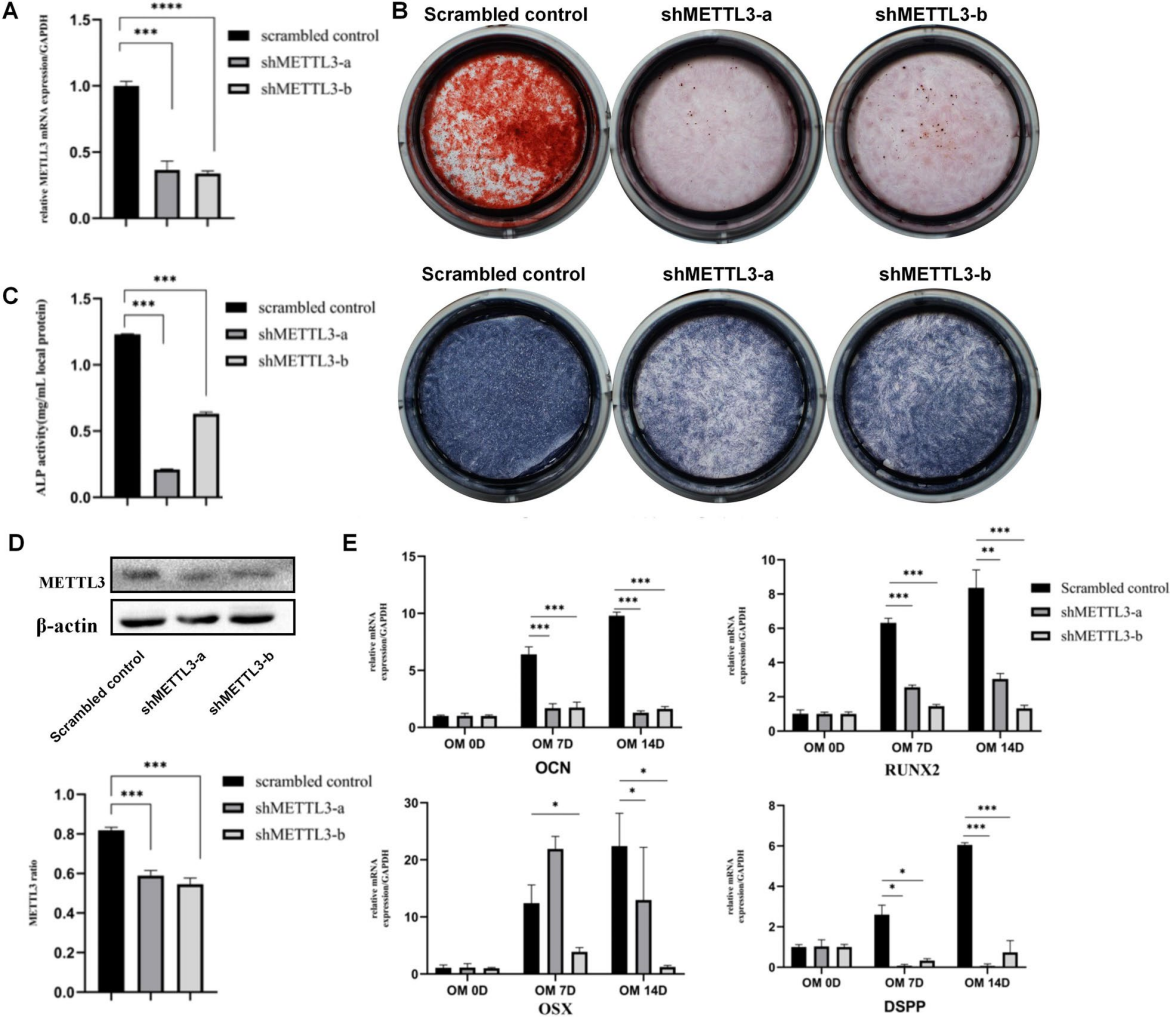
Knockdown of METTL3 inhibited the dentinogenesis differentiation of DPSCs. **A** METTL3-kncokdown lentiviral were used to knockdown METTL3 (shMETTL3-a, shMETTL3-b) in DPSCs. The knockdown efficiency of METTL3 gene expression in DPSCs, detected by real‐time RT‐PCR (n = 3). **B** Alizarin red S staining on 14 d, and C)ALP staining on 7 d of DPSCs cultured in OM (n = 3), **C** The quantitative analysis and staining of ALP activity (n = 3). **D**The knockdown efficiency of METTL3 gene expression detected by western blotting (n = 3). **E** Expression levels of dentinogenesis differentiation-related genes, OCN, RUNX2, OSX, DSPP, were measured by real‐time RT‐PCR on 0, 7 and 14 days after mineralization induction (n = 3). *P < 0.05, **P < 0.01, ***P < 0.001

DPSCs were cultured in vitro and impaired the METTL3 expression level with METTL3-kncokdown lentiviral (shMETTL3-a, shMETTL3-b). DPSCs cultured with scrambled control lentiviral were set as control. The knockdown efficiency was verified with real‐time RT‐PCR and western blotting (Fig. 4A, D). Alizarin red S staining at 14d displayed that there were fewer mineralized nodules in the METTL3-kncokdown group compared with scrambled control group (Fig. 4B). The ALP staining and quantitative analysis also showed that the ALP activity of DPSCs was also lower in METTL3-knockdown group (Fig. 4C). In addition, real time RT‐PCR was performed on 0d, 7d and 14d to detect the mRNA expression of dentinogenesis differentiation related genes, including *OSX, OCN, RUNX2* and *DSPP*. In according with alizarin red S staining and ALP activity, these genes were significantly down-regulated in knockdown groups (Fig. 4E). FTO inhibitor, meclofenamic acid (MA), was used to inhibit the FTO activity of DPSCs. The results showed that high concentration of the MA significantly reduced DPSCs proliferation and dentinogenesis differentiation (Additional file 1: Fig. S1A–D). This effect of FTO was against to the increase of m^6^A methylation, while the effect of METTL3 in accordance with the dynamic change of m^6^A methylation during DPSCs dentinogenesis differentiation. Thus, METTL3 was selected for further study.

**Fig. 5:**
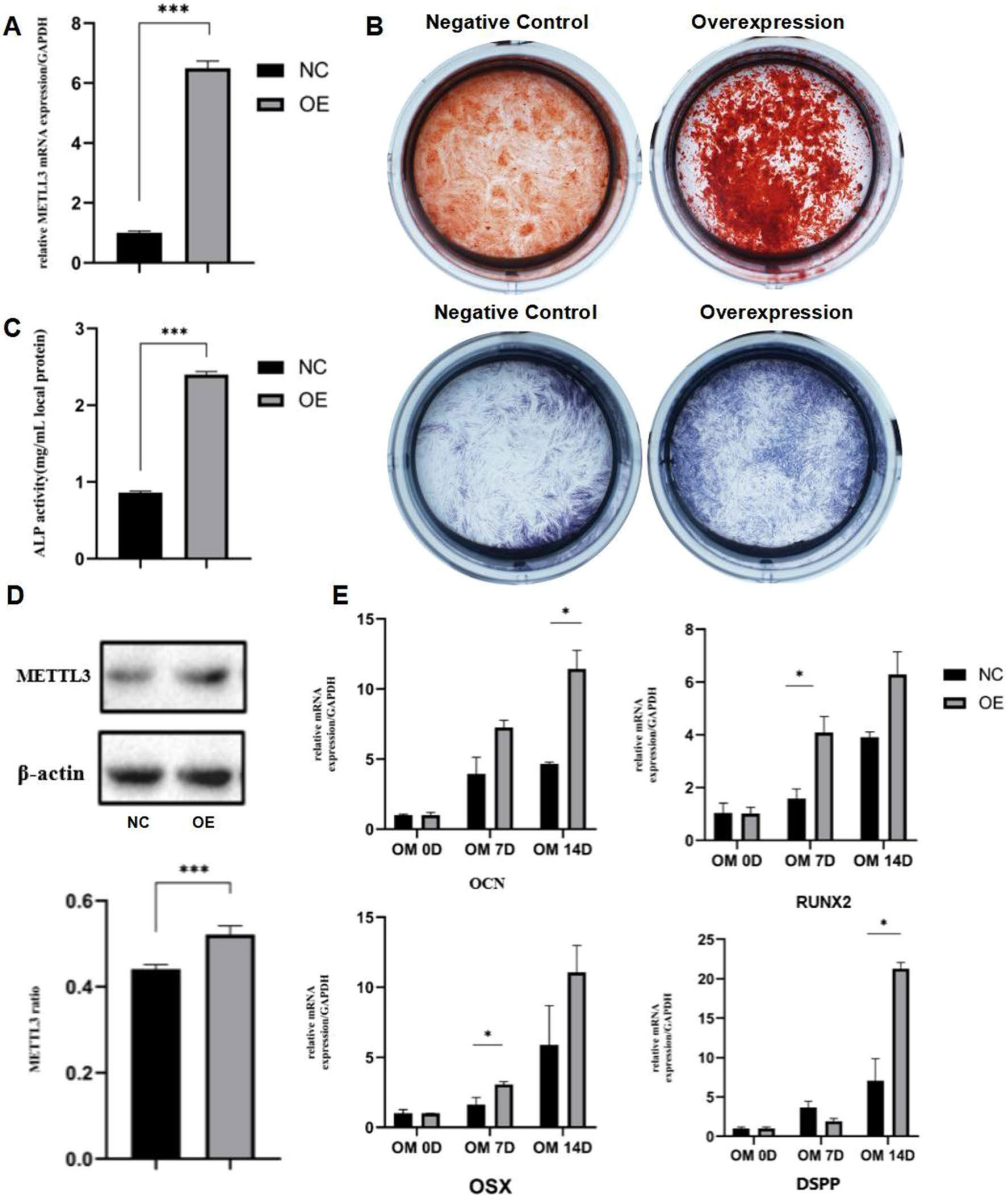
Overexpression of METTL3 promoted the dentinogenesis differentiation of DPSCs. **A** METTL3 overexpression lentiviral were used to infect DPSCs in order to over-express METTL3 (OE). The overexpression efficiency of METTL3 gene expression in DPSCs, detected by real‐time RT‐PCR (n = 3). **B** Alizarin red S staining on 14 d, and **C** ALP staining on 7 d of DPSCs cultured in OM (n = 3), C)The quantitative analysis and staining of ALP activity (n = 3). **D** The overexpression efficiency of METTL3 gene expression detected by western blotting (n = 3). **E** Expression levels of dentinogenesis differentiation-related genes, OCN, RUNX2, OSX, DSPP, were measured by real‐time RT‐PCR on 0, 7 and 14 days after mineralization induction (n = 3). *P < 0.05, **P < 0.01, ***P < 0.001

Then, METTL3 was up-regulated in DPSCs by METTL3-overexpression lentiviral successfully (OE), and DPSCs cultured with scrambled control lentiviral were set as control (NC). The overexpression efficiency was verified by real time RT‐PCR and western blotting (Fig. 5A, D). Alizarin red S staining showed that there were more mineralized nodules in the METTL3-overexpression group than the negative control group (Fig. 5B). The staining and quantitative analysis also displayed that ALP activity of METTL3-overexpression group was higher than NC group (Fig. 5C). Similarly, the mRNA expression of dentinogenesis differentiation related genes were increased in DPSCs after METTL3 overexpression (Fig. 5E), further indicating that METTL3 promoted DPSCs dentinogenesis differentiation in vitro. This results indicated that METTL3 participated in dentinogenesis differentiation via RNA m^6^A modification.

### METTL3 mediated RNA m^6^A methylation regulated GDF6 and STC1 mRNA stability

**Fig. 6:**
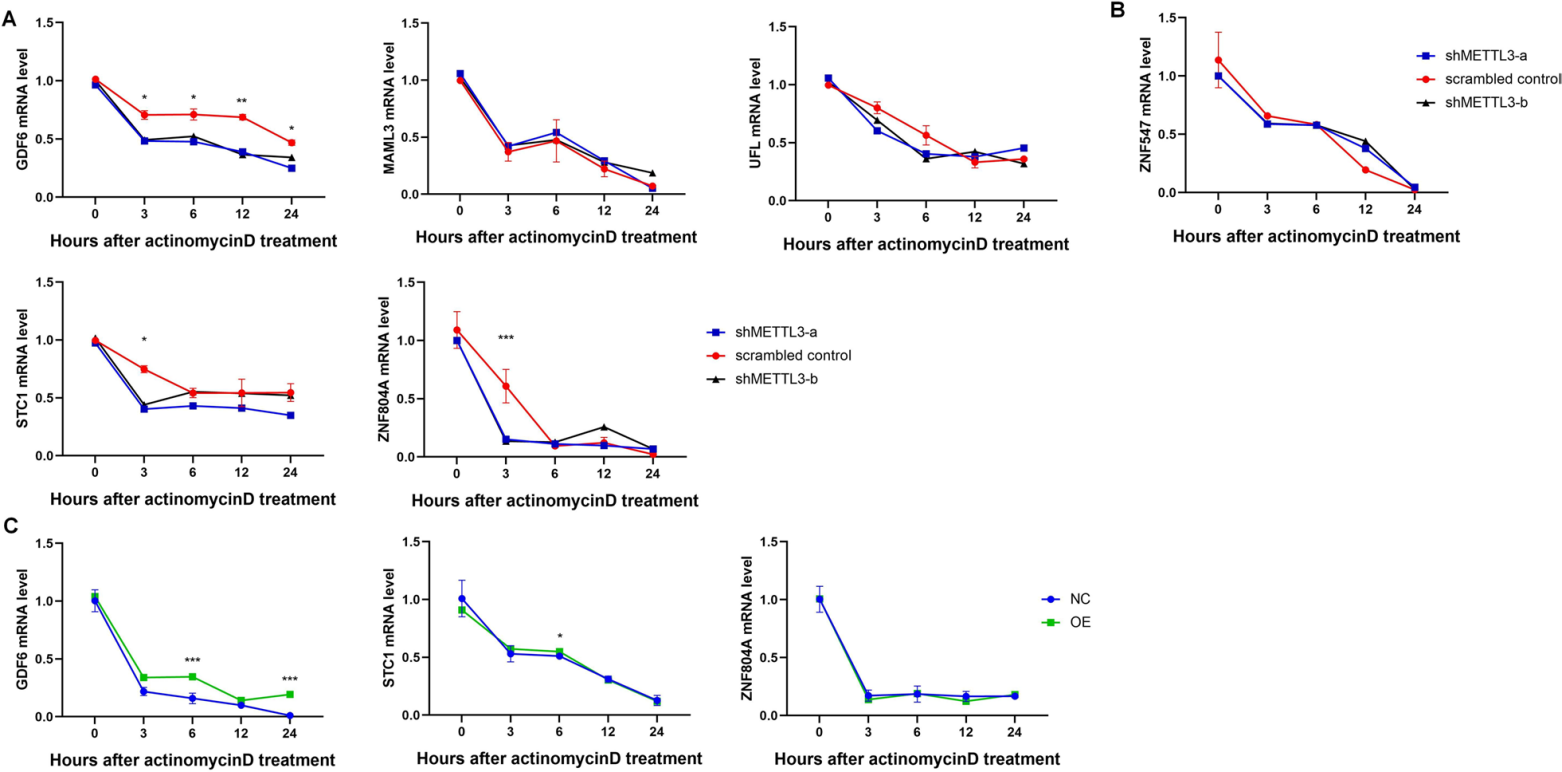
The mRNA stability of genes in DPSCs. **A** The mRNA stability of genes with hypermethylation and up-transcription, and **B** gene with hypermethylation and down-transcription in METTL3 knockdown DPSCs. **C** Genes with hypermethylation and up-transcription in METTL3 overexpression DPSCs (n = 3).*P < 0.05, **P < 0.01, ***P < 0.001

The genes with hyper-methylation peaks and up-transcription (*GDF6, MAML3, MICALCL, OXCT2, STC1, UFL1, ZNF441,* and *ZNF804A)* were annotated with chipseeker (Additional file 3: Table S2) [27]. Among them, *GDF6, MAML3, STC1, UFL1,* and *ZNF804A* had 3’UTR with m^6^A methylation. As 3’UTR is closely related to mRNA stability, m^6^A modification might control genes with methylated 3’UTR to effect dentinogenesis. So the mRNA stability analysis were carried out with actinomycin D. Compared with control groups, the mRNA level of *GDF6, STC1, and ZNF804A* decreased in METTL3-knockdown group (Fig. 6A)*.* While, the mRNA level of *MAML3* and *UFL* remained unchanged. Besides, mRNA level of hyper-methylated and down-transcripted gene *ZNF547*, which also had clustered m^6^A modification in 3’UTR, changed without significantly (Fig. 6B).

To confirm the effort of METTL3 in regulation of the mRNA stability, actinomycin D was also used in METTL3 overexpression group. The mRNA level of *GDF6* and *STC1* increased, and the level of *ZNF804A* mRNA changed without significantly (Fig. 6C). The results suggested that METTL3-mediated m^6^A involved in dentinogenesis differentiation by enhancing the mRNA stability of *GDF6* and *STC1*.

### Overexpression of METTL3 promoted the tertiary dentin formation in vivo

**Fig. 7:**
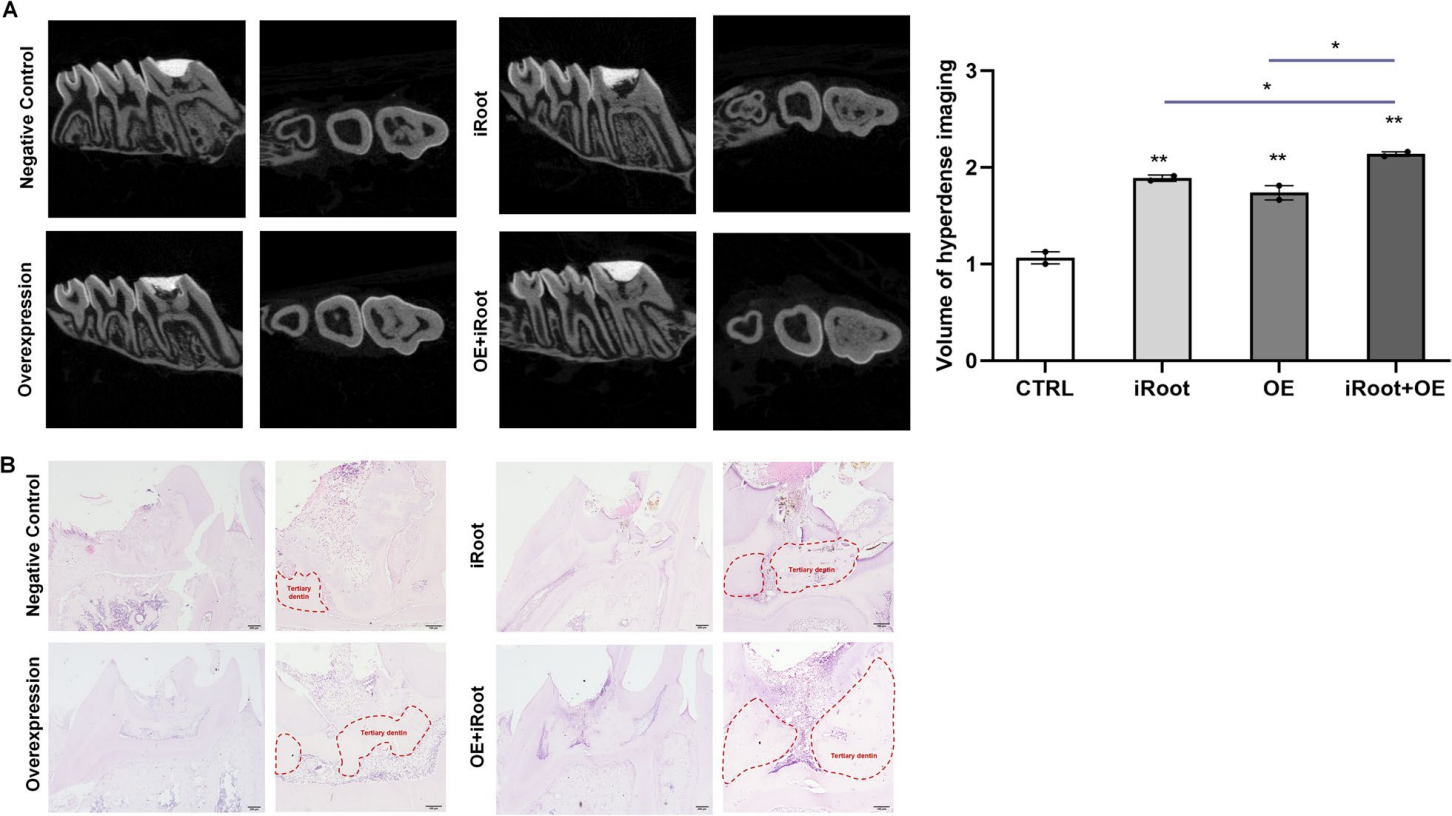
METTL3 promoted the formation of tertiary dentin. **A** Micro CT analysis of rat maxillae after pulp capping (n = 12). **B** The structure of mineralized tissue was detected by HE staining (n = 12). *P < 0.05, **P < 0.01

To explore the potential application of METTL3 in VPT, direct pulp capping model was established with rat first molars. 30 days after operation, the maxillae were scanned by MicroCT and found that there were hyper-dense imaging in pulp chambers beneath the dentin around the pulp exposure point (Fig. 7A). There were barely hyper-dense imaging found in control group. In contrast, the volume of hyper-dense imaging in METTL3 OE + iRoot group was the highest, followed by iRoot group and METTL3 OE group. While, there were also more hyper-dense imagings in METTL3 OE group than the control group. These hyper-dense imagings suggested that there might be tertiary dentin formed after pulp capping with METTL3 overexpression lentiviral. The following HE staining showed that there were mineralized tissue in the pulp chamber beneath dentin around the pulp exposure point, with the dentinal tubules lined up in disorder and cells rounded irregularly (Fig. 7B), indicating that application of METTL3 in VPT promoted the tertiary dentin formation.

## Discussion

RNA m^6^A methylation is the most abundant mRNA internal modification in eukaryotic cells. METTL3, as the methyltransferases, one of the essential regulators, involves in the process of dentin formation and root development. In this study, we established the dynamic RNA m^6^A methylation profile in dentinogenesis differentiation and confirmed the essential role of METTL3 in the DPSC dentinogenesis differentiation.

Epigenetic regulation changes the expression level and function of genes without changing the sequences [28]. It involves in many biological processes, such as embryonic development, bone homeostasis, and the fate of stem cells [29]. RNA m^6^A modification could also effect the fate of stem cells by post-transcriptional regulation. Previous study explored the m^6^A methylation pattern in rat cortex after traumatic brain injury (TBI) by MeRIP-seq [30]. They compared the methylation levels of rat cortex among the TBI group and sham group and found that there were significant m^6^A methylation changes after TBI. It suggested that the modification of m^6^A methylation involved in the development of diseases and injuries. Latest research used normal human oral epithelial cells (HOEC), precancerous dysplastic oral keratinocyte (DOK) cells, and oral squamous cell carcinoma (SCC-9) cells to explore the m^6^A modification in oral precancerous cells [31]. The significant m^6^A peak changes in DOK and SCC-9 cells demonstrated that the characteristics of m^6^A modification indicated the development phase of carcinogenesis. In the present study, we established the dynamic profile of RNA m^6^A methylation during dentinogenesis differentiation with MeRIP-seq for the first time. We found that the modification of m^6^A methylation changed significantly during this process. It suggested the potential regulatory role of RNA m^6^A modification in DPSCs fate determination.

The dynamic characteristic of RNA m^6^A modification relies on the regulation of RNA methyltransferases. Methyltransferases, METTL3, METTL14 and WTAP, form complexes to perform catalytic functions together. While, METTL14 was reported no catalytic activity for methylation, but it could stabilize the binding of METTL3 and target mRNA. [32] Previous studies decreased m^6^A methylation in BMSCs by knockdown METTL3 successfully [33, 34]. With the inhibition of m^6^A modification, the osteogenic differentiation in BMSCs was impaired. It was reported that knockdown of METTL3 inhibited the DPSCs proliferation, migration, and caused shortened molar roots and defected dentin formation in mice [19]. METTL3 inhibition also effected DPSCs senescence and apoptosis, further resulting in the reduction of regenerative potential in mature teeth [18]. In our research, we also inhibited METTL3 in DPSCs and proved that impairing METTL3 reduced the dentinogenesis differentiation. The result further confirmed the role of METTL3 in regulation of DPSCs fate determination. We also found that the enrichment of demethylase FTO in DPSCs dentinogenesis was against to the increase of m^6^A modification. It was demonstrated that FTO was up-regulated during the osteogenic differentiation in human MSCs, by mediating m^6^A demethylation of osteoporosis biomarker in a YTHDF1-dependent manner. [35] It suggested that FTO maybe promoted the dentinogenesis differentiation through other ways.

METTL3 has the ability to distinguish and target m^6^A sites, enhancing the RNA m^6^A modification and affecting the metabolism of RNA in many ways [36]. It can read, regulate m^6^A marks near 3’UTR of mRNA and promote mRNA translation [37]. The 3’UTR of mRNA serve vital roles in post-transcriptional gene expression, regulating mRNA stability, translation, and localization. [38, 39] Stress-induced rG4s were enriched in mRNA 3’UTR regions and enhanced mRNA stability in human osteosarcoma (U2OS) cells. [40] It was observed that 3’UTR-rG4-bearing mRNAs had half-lives under stress, showing higher mRNA stabilities. Latest research also reveled that RUNX2 mRNA had a m^6^A methylated target at 3’UTR [41]. METTL3 mediated m^6^A modification at the region to enhance the stability of RUNX2 mRNA, promoting the osteogenic differentiation of BMSCs. In our study, most of the hyper-methylation peaks were also annotated at 3’UTR after dentinogenesis differentiation. And knockdown METTL3 significantly decreased the *GDF6* and *STC1* mRNA stability, while it in METTL3-overexpression group increased. These results demonstrated that METTL3 promoted the dentinogenesis differentiation by enhangcing the stability of *GDF6* and *STC1* mRNA through methylated their 3’UTR regions.

Growth differentiation factor 6 (GDF6) is a member of bone morphogenetic protein (BMP), also known as BMP13. Previous study announced that GDF6 induced mesenchymal stem cells (MSCs) to differentiate into a nucleus pulposus cell-like phenotype [42]. It was also reported that GDF6 promoted the ligament tissue differentiation in MSCs [43]. Stanniocalcin1 (STCl), a member of the calcitonin family, is first found in bony fish and plays a role in regulating calcium and phosphorus balance. At present, it is known that STCl is necessary for calcium regulation and phosphorus balance and cell metabolism in kidney and small intestine [44]. While, the mechanism of GDF and STC1 regulate the dentinogenesis differentiation still need further study.

In clinical treatment, the success of VPT is closely related to the pulp capping agents. The agents should have the advantages of easy operation, good bio-compatibility and x-ray resistance. Previous study used chitosan as a scaffolding to carry human endometrial stem cells (EnSCs) in VPT. [45] Chitosan is bio-compatible, bio-degradable, and non-toxic, which is capable to use in medical applications. Gelatin sponge was also reported to be used in rat direct pulp capping models. [46, 47] In our study, we applied METTL3 with GSs in VPT and found that the treatment promoted the tertiary dentin formation. Although the GS is bio-compatible, bio-degradable, and widely used in dental treatment, it has poorly x-ray resistance. Thus, we are still making efforts to find a better agent to carry METTL3-overexpression lentiviral to improve our model.

## Conclusion

The present study established the dynamic m^6^A modification profiles in DPSCs dentinogenesis differentiation, and revealed that METTL3 promoted DPSCs differentiation through enhancing the mRNA stability of *GDF6* and *STC1*. The application of METTL3 also promoted tertiary dentin formation, which provided new insights into VPT. An in-depth understanding of the epigenetic regulation of DPSC fate will facilitate the search for capping agents that promotes DPSCs dentinogenesis differentiation, which is a promising direction in the search of clinical treatment for functional dentin reconstruction.

## Supplementary Information

**Additional file 1.** Supplementary metarials.

**Additional file 2.** Western blotting with membrane edges visible.

**Additional file 3. Table S2.** Peaks annotationin of hyper-methylated and up-transcripted genes.

## Data Availability

Raw data were generated at State Key Laboratory of Oral Diseases & National Clinical Research Center for Oral Diseases, West China Hospital of Stomatology, Sichuan University. Derived data supporting the findings of this study are available from the corresponding author, Mian Wan, on request.
