# Supplementary material for: METTL3 enhances dentinogenesis differentiation of dental pulp stem cells via increasing GDF6 and STC1 mRNA stability
Source: BMC Oral Health. 2023 Apr 11;23:209. doi: 10.1186/s12903-023-02836-z (PMC10088233; doi:10.1186/s12903-023-02836-z)
Supplement: Supplementary file 1 — Additional file 1. Supplementary metarials. [file 12903_2023_2836_MOESM1_ESM.docx]

**Supplementary Materials**

| **Gene** | **Primers** |
| --- | --- |
| GAPDH | F 5’ GGAGCGAGATCCCTCCAAAAT 3’ |
|  | R 5’ GGCTGTTGTCATACTTCTCATGG 3’ |
| RUNX2 | F 5’ CCTTTACTTACACCCCGCCA 3’ |
|  | R 5' GGATCCTGACGAAGTGCCAT 3’ |
| OCN | F 5’ ATTGTGGCTCACCCTCCATC 3’ |
|  | R 5’ CCAGCCTCCAGCACTGTTTA 3’ |
| OSX | F 5’ TCTGCGGGACTCAACAACTC 3’ |
|  | R 5’ TAGCATAGCCTGAGGTGGGT 3’ |
| DSPP | F 5’ ATATTGAGGGCTGGAATGGGGA 3’ |
|  | R 5’ TTTGTGGCTCCAGCATTGTCA 3’ |
| METTL3 | F 5’ GAGGAGTGCATGAAAGCCAG 3’ |
|  | R 5’ GGCCTCAGAATCCATGCAAG 3’ |
| METTL14 | F 5’ GACGGGGACTTCATTCATGC 3’ |
|  | R 5’ CCAGCCTGGTCGAATTGTAC 3’ |
| FTO | F 5’ AGACACCTGGTTTGGCGATA 3’ |
|  | R 5’ CCAAGGTTCCTGTTGAGCAC 3’ |
| ALKBH5 | F 5’ ACCCCATCCACATCTTCGAG 3’ |
|  | R 5’ CTTGATGTCCTGAGGCCGTA 3’ |
| GDF6 | F 5’ CGATCTCTCGCACACTCCTC 3’ |
|  | R 5’ GCCCACCAGCTCTTCTTTGT 3’ |
| STC1 | F 5’ AACCAGTAACGTGCTGCTCA 3’ |
|  | R 5’ TAAGTTTGGGGACCTGGGGA 3’ |
| MAML3 | F 5’ GTTTCAAGGTTCTCCCCAGGAT 3’ |
|  | R 5’ GGTGCTATAAGGGGCCAGTC 3’ |
| UFL1 | F 5’ CAGTTCCTCCGCGTCTACTG 3’ |
|  | R 5’ CGCCAACCGCCTAATCTCTT 3’ |
| OXCT2 | F 5’ ACAAAGGACAACACCCCCAA 3’ |
|  | R 5’ CCTCAGCGTCAGCTCTTTCT 3’ |
| ZNF441 | F 5’ GAGGACCCTTTACCCAGACTC 3’ |
|  | R 5’ GAGATGAACGACCCATGAGGA 3’ |
| ZNF804A | F 5’ ACCAGTAGGACCGAGGCTTT 3’ |
|  | R 5’ CGGAAGCTGGAATGATTGGC 3’ |
| RAB40A | F 5’ AGAAAGTGTGACGTGGGTGAA 3’ |
|  | R 5’ CCTGTTCCCAAAAACCACAGTC 3’ |
| ZNF547 | F 5’ TCTCTTCCCTGGCTGGACTTG 3’ |
|  | R 5’ ACATCACGGTACAGCAATCTCT 3’ |
| ZNF14 | F 5’ GATTGCGTTTCACCTGCTCC 3’ |
|  | R 5’ CTGAGTCCATTTCCCAGCGT 3’ |

Supplementary Table 1. Primers used for qRT-PCR.


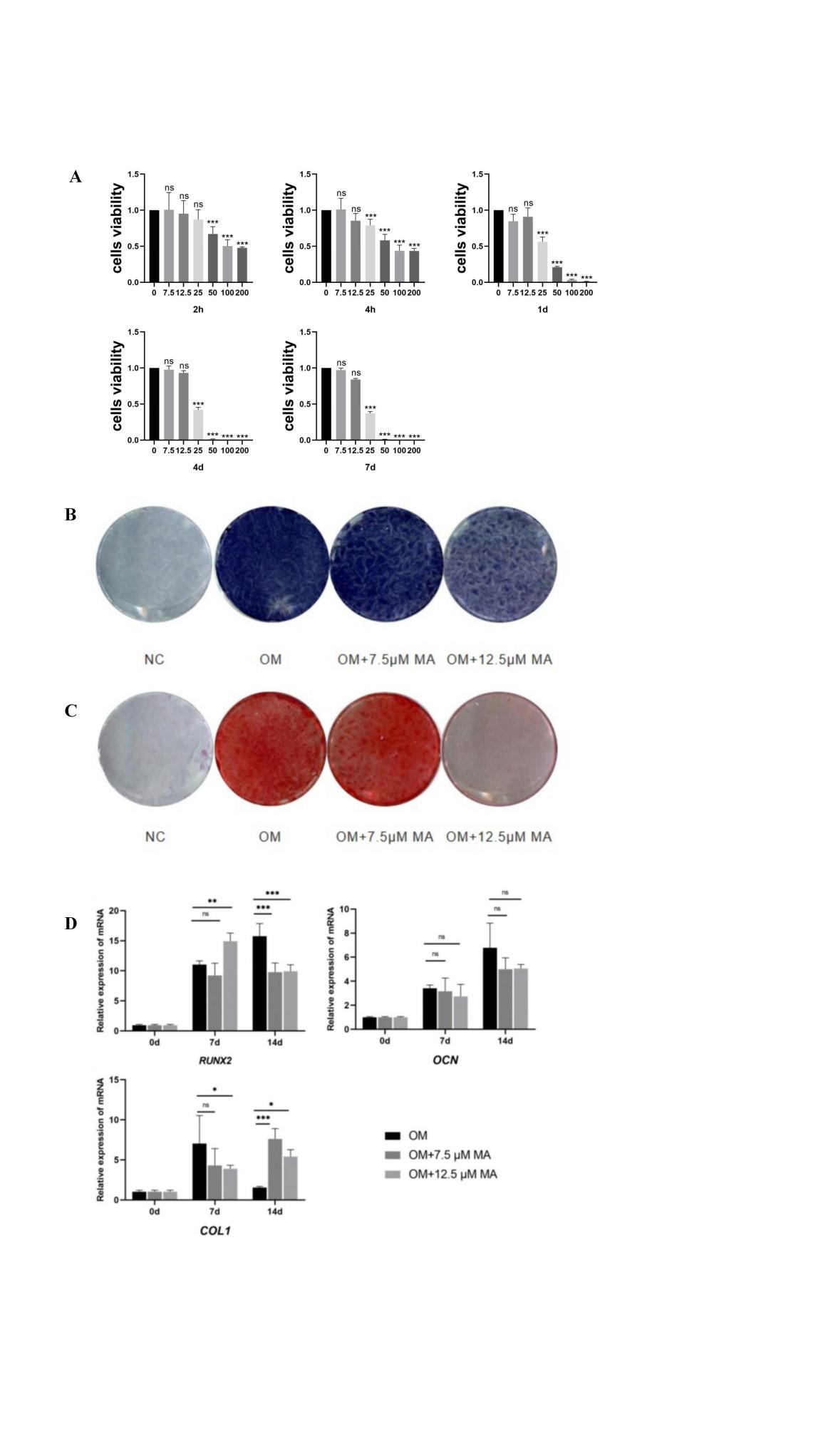


Supplementary Figure 1. FTO inhibitor reduced the odontoblast differentiation of DPSCs. A)Cell viability of DPSCs after 6 different concentrations of MA treated, and 7.5μM MA and 12.5μM MA group were selected for following experiments (n = 5). B)ALP staining on 7 d, and C)Alizarin red S staining on 14 d of DPSCs cultured in OM (n = 3). D)Expression levels of odontoblast differentiation related genes, RUNX2, OCN and COL1, were measured by real‐time RT‐PCR on 0, 7 and 14 days after mineralization induction with MA treatment (n=3). **P* < 0.05, ***P* < 0.01, ****P* < 0.001
