# Supplementary figures and images for: METTL3 enhances dentinogenesis differentiation of dental pulp stem cells via increasing GDF6 and STC1 mRNA stability
Source: BMC Oral Health. 2023 Apr 11;23:209. doi: 10.1186/s12903-023-02836-z (PMC10088233; doi:10.1186/s12903-023-02836-z)

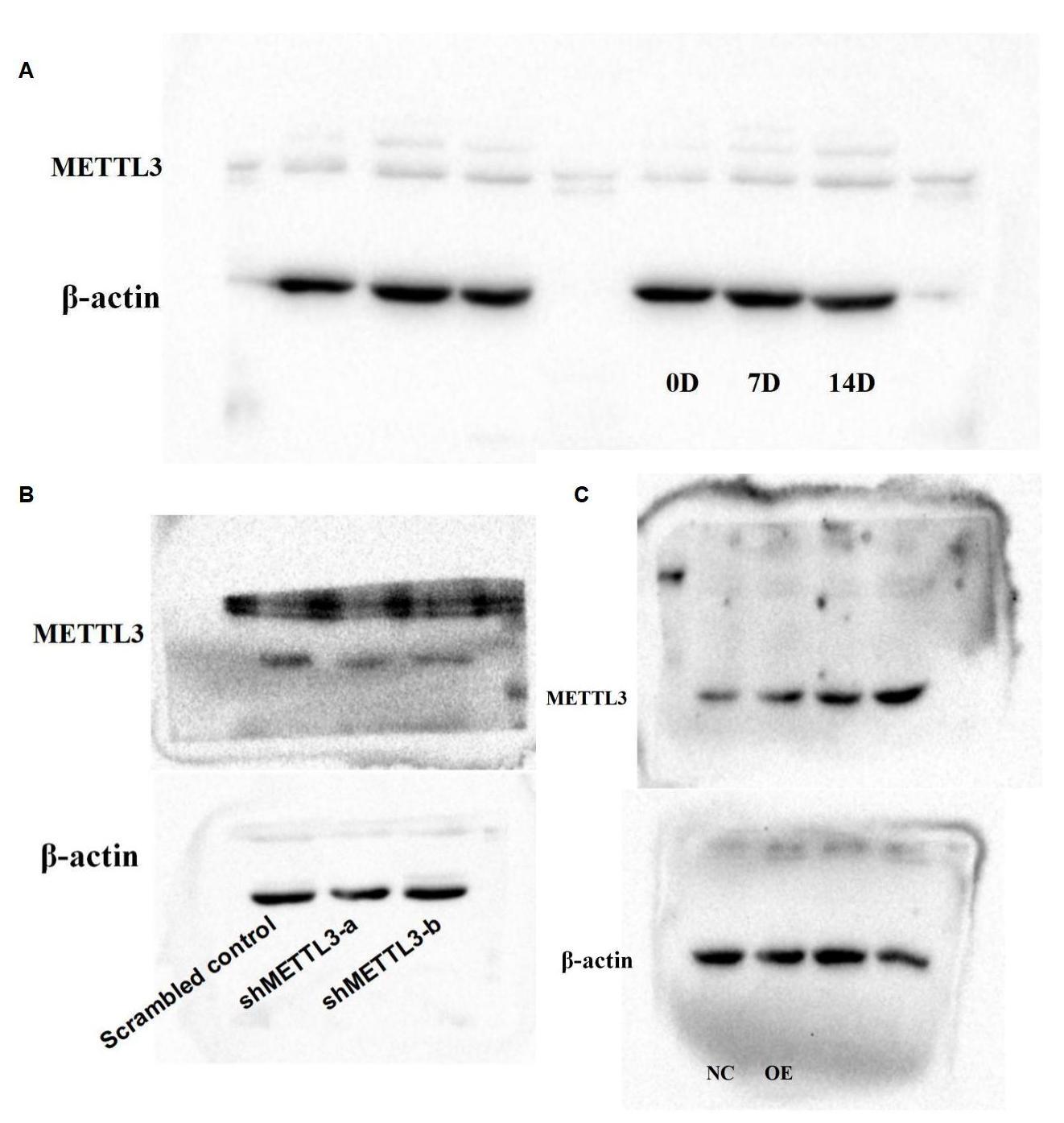

Supplement: Supplementary file 2 — Additional file 2. Western blotting with membrane edges visible. [file 12903_2023_2836_MOESM2_ESM.jpg]
